# A New Paradigm of Primary Health Care in Kazakhstan: Personalized, Community-based, Standardized, and Technology-driven

**DOI:** 10.5195/cajgh.2014.186

**Published:** 2014-09-29

**Authors:** Almaz Sharman

**Affiliations:** Co-Founder and CEO, HealthCity LLP, Almaty, Kazakhstan; President, Academy of Preventive Medicine of Kazakhstan

**Keywords:** HealthCity, SmartHealth, Health and wellness, Healthcare

## Abstract

This paper discusses the need for change from Kazakhstan’s current disease-centric healthcare paradigm to a new primary health and wellness-centric health care paradigm, technology-driven and based on personal relationships within a social context. While many different papers have been published about the importance of prevention and primary health care, few have focused on healthcare transition in Kazakhstan or other countries in Central Asia. The WHO’s historic 1978 Alma-Ata Declaration signed in Kazakhstan promoted the centrality of primary care to the provision of effective, efficient, and equitable health services. Modern technologies such as the Internet, social media, and portable medical devices democratize medicine, providing great opportunities to rethink the Alma-Ata Declaration and reinvent primary health care on an entirely new platform that is knowledge-based and technology-assisted. The new paradigm suggested for the future development of health in Central Asian region emphasizes personal relationships and encourages sustainable solutions created by communities. This paper also introduces HealthCity, a new project in Kazakhstan aiming at introducing private, community-based and standardized primary healthcare that is driven by SmartHealth innovative technology.

## Current Healthcare Paradigm is Disease-centric

Because of vast hydrocarbon resources available in Kazakhstan, this Central Asian country experienced tremendous growth and rapid economic expansion in recent years. Over the 20 years of independence, transformation of Kazakhstan’s economy, lifestyle, and social system have influenced the burden of both infectious and chronic disease and increased demand for health services. 1 Following the break-up of the Soviet Union, Kazakhstan experienced a dramatic increase in tuberculosis and other infectious diseases. 2 On the other hand, the health of population in Kazakhstan is characterized by high prevalence of cardiovascular disorders, cancer, diabetes, and other non-communicable diseases. Combined with a rapidly aging population and an outdated healthcare infrastructure, Kazakhstan’s healthcare provider network is facing challenges associated with country’s healthcare transition. Significant changes will be required, at both the national and regional levels, to meet the current and future health needs of its population.

In Kazakhstan, the government owns or has control over approximately 80% of medical institutions, which include more than 1,000 hospitals and 3,400 ambulatory care clinics. Although public healthcare spending was forecasted to reach $6 billion in 2014 and expected to grow at a 4-year Compund Annual Growth Rate (CAGR) of 13.4% to $9.1 billion in 2017, it may prove difficult to absorb and efficiently allocate the government’s increased levels of investment given constraints such as shortfalls of skilled healthcare professionals. 3

Public and private health expenditures in Kazakhstan exceed 4% GDP, of which 2.6% from public sources. 4 Despite such investments, Kazakhstan’s healthcare is characterized by insufficient access to the latest technology and slow integration of evidence-based clinical care practices. Addressing the need for raising additional healthcare funds, the government of Kazakhstan has made a decision to introduce national mandatory health insurance system starting 2017. Based on previous personal communications with Ms. Tamara Duisenova, the Minister of Health and Social Services, funding for insurance will be generated from three sources: (1) government guaranteed healthcare benefits package; (2) individual co-payment, and (3) additional payroll taxes. While the government plays a dominating role in healthcare, the private sector (households and private health insurance) contributes around 34 percent of total health expenditures. Households contribute most private funds on an out-of-pocket basis. 5

Inability of the state-run health care system to meet the growing healthcare demands of Kazakhstan population has forced people of Kazakhstan to turn to a growing array of private health services that operate based on fee for service system. As a result, a dual health care picture has emerged in Kazakhstan: an inefficient old state run system; and a second, loosely regulated market–based private system that offers competitive solutions to deficiencies of the government system.

## Extensive Hospital Infrastructure

In most countries, the current healthcare paradigm can be described as disease-centric. Medical establishment and the general public often believe that hospitals and efforts to treat diseases lead to better healthcare and more active and productive lives. Investing in hospitals and in-patient care still dominates the healthcare of Kazakhstan. An extensive hospital infrastructure and a neglect of primary health care is one of the legacies of the Soviet health system. Even though Kazakhstan has undertaken many health related reforms over the last 20 years, the inherited public health and medical infrastructure still determines provision of care.

Current public allocations for in-patient services in Kazakhstan have doubled within the past 5 years. According to a report by Oxford Policy Management (OPM), a large number of hospitals in Kazakhstan still treat patients on an in-patient basis, despite the fact that many of them could be effectively treated on an outpatient basis. Few years ago, such cases collectively amounted to around 15% of hospital discharges. Consequently, a significant portion of healthcare resources were spent on in-patient services instead of funding the primary health care. Overall, less than 5% of the national health expenditures are allocated to support primary healthcare and disease prevention. 6 This is especially true in the city of Astana, the capital of Kazakhstan, where a dozen of new in-patient facilities (tertiary care hospitals) were built during the last 10 years.

Not only are too many hospitals being built, they are also becoming fancier and much more expensive to build, equip, and maintain. If in the ancient world marble was used to build temples and pyramids and in medieval times marble was used to build castles and churches, the 20th century used it to outfit banks and offices. Where does the marble go now? …To build hospitals.

Equipping the tertiary care facilities introduces extra costs that burden the healthcare system in Kazakhtan. While most consumer technologies such as smartphones and tablet PCs are becoming more sophisticated but cheaper and easier to use, medical technologies are moving in the opposite direction. They have become more expensive, complicated, and often very intimidating to patients.

## Permanent Battle Against Diseases

We are in a state of permanent battle with our eternal enemy – diseases and maladies. The enemy gains more power by engaging weapons of mass destruction - debilitating chronic illnesses such as heart disease, dementia, diabetes and cancer, as well as emerging and re-emerging infections. Inventing new drugs and medical technologies has become increasingly difficult and resource-intensive. The fruits of medical knowledge that grew at the bottom of the tree of knowledge have already been picked - all that are left are the ones on top, demanding more efforts and resources. 7 Modern medicine deals with almost 14,000 diseases and operates with about 6,000 different types of medications and 4,000 types of medical procedures. 8 Despite having this rich arsenal of treatments, we are consistently losing our battles against disease because our curative strategies are often inefficient and costly. Medical mistakes, that are especially common in tertiary care facilities kill enough people around the world each day to fill many jumbo jets. 9

250 years ago, French Enlightenment writer François Voltaire wrote, “Doctors prescribe medicine of which they know little, to cure diseases of which they know less, in human beings of which they know nothing.” 10 We are still focusing on inventing new drugs and medical technologies, but what we really do is fighting diseases at the point where the body has almost been conquered by the disease. Is this really the right direction that is being taken by our healthcare systems?

An alternative to this is a change from the current paradigm that is diseases-centric to a new paradigm, the one that is health and prevention-centric, technology-driven and is based on personal relationships within a social context.

## Primary Care is a Backbone of Healthcare System

In 1946, the World Health Organization defined health as: “… a state of complete physical, mental and social well-being and not merely the absence of disease or infirmity.” 11 Despite tremendous gains in life expectancy achieved in the 20^th^ century, poor physical, mental and social well-being continues to burdensome and costly issue throughout the world in the 21^st^ century. One of the key contributing factors to this paradox is that we keep forgetting the key role of disease prevention and early detection, which is an essence of primary health care.

The WHO’s historic Alma-Ata Declaration of 1978 promoted the centrality of primary care to the operation of effective, efficient, and equitable health services. 12 While many countries adopted the Declaration, primary health care has been neglected and remains underfunded in many countries around the world including Kazakhstan. A significant portion of national healthcare resources go towards in-patient services instead of funding primary health care and shifting the balance in favor of the latter. 13

An inevitable result of such policies is an increase in healthcare utilization, increasing burden of chronic disease care, and subsequently, rising healthcare costs. Aging populations, environmental issues, as well as emerging and re-emerging infections make it even more difficult to contain healthcare costs. Focusing on primary care and disease prevention is one of the key strategies that can be effectively implemented to contain increasing healthcare costs.

Primary health care is not just the first line of defense, but an effective gateway that facilitates communication with the various components of healthcare system. If country’s primary health care system is strong, hospital admissions are typically reduced by 40% and health care costs are reducted by 30%. Previous research demonstrated that around 70% to 80% of health-related episodes in an individual’s life can be identified by general practitioners at a primary care level outside hospitals. 14

Primary care doctors are not superstar surgeons, but their role is crucial. They are the ones who protect consumers of healthcare from getting ill and help them navigate in the ocean of medical knowledge and healthcare institutions. Their mission is to organize the full range of healthcare services: from individual to household to hospitals and from disease prevention to treatment and rehabilitation.

In many countries around the world primary care doctors that are considered to be “Cinderellas” of healthcare. They don’t share the glory of sophisticated medical technologies, their salaries are fractions of what surgeons get, and their role is not recognized. We need to empower them with knowledge and technology, while motivating and properly compensating them for their hard work.

## Community-based and Technology-driven Healthcare

One of the key ways to bridge multiple disciplines, professions, and approaches to healthcare is to work towards developing patient centered personalized medicine. This can be achieved through practicing primary health care that is community-based, utilizes technology, and incorporates patient wishes and values. The seventh tenet of the Alma-Ata Declaration says that, “The people have the right and duty to participate individually and collectively in the planning and implementation of their health care.” The Declaration also implies that “primary health care …requires and promotes maximum community and individual self-reliance and participation…” 12

Primary health care emphasizes local ownership and encourages sustainable solutions created by communities. 15 The team-based approach that is essential in addressing the healthcare needs of a person and community. 16 The key question is how to engage all the players – consumers of healthcare, primary care doctors and nurses – not just the specialty doctors and hospitals.

Modern technologies provide great opportunities to reinvent primary health care on an entirely new platform. Internet and social media as well as portable EKG and ultrasound machines, iPhone medical apps and devices democratize medicine making knowledge and technology available to the doctors and consumers. 7,17 Dr. Eric Topol described this trend as, “creative destruction of medicine.” 10

Technology creates a new primary health care environment that is more efficient and knowledge-based. In average, every day we spend 5 hours on Internet, and 77% of Internet users rely on this technology to search for medical information. In 2013, Google registered 30 billion searches for medical information associated terms. When analyzed historical query-based flu estimates for different countries and regions compared against official influenza surveillance data, it was determined that estimates based on Google search queries about flu are very closely matched to traditional flu activity indicators. 18

Electronic patient records help to reduce unnecessary human intervention and free time for cost-effective and high quality services. Specialized social networks for healthcare professionals such as Doximity, enable primary care doctors to easily get on the same page with specialists and more effectively collaborate by sharing notes, records and other information. Technology-driven primary health care is an effective way to build collaborations among different healthcare sectors.

## The Need for Patient-centered and Personalized Care

According to a population-based representative household health survey conducted in 2012 by the Academy of Preventive Medicine of Kazakhstan, more than 30% residents of Almaty, Kazakhstan aged 25–44 prefer seeking medical care in private healthcare facility. More than 40% among them believe that they can be harmed when treated in the government ambulatory and in-patient medical facilities. 19 Disadvantages of government based medical system in Kazakhstan include long waiting lines, need to rely on friends and family to know which doctor is good, inability to get consistent diagnosis if you go to more than one doctor, poor medical record retrieval system, and many others. These challenges translated into unsatisfied consumers, both on individual and community levels. Dissatisfied with poor quality of medical services, many Kazakh citizens turn to medical tourism to solve their healthcare issues. A study conducted a few years ago by Harvard Medical International reported $200–250 million that channeled annually from Kazakhstan to overseas to cover the costs of medical care. 20 There is an opportunity to capture those who travel abroad for medical care by providing high quality medical services at home.

With the emerging middle class in Kazakhstan, we are witnessing the evolvement of healthcare market, with consumers becoming interested in better healthcare. These consumers will ultimately want to be empowered to make their own health decisions and be in the center of their care, with access to high quality services. In the opinion of the author, personalized medicine is when doctors practice with the consumer at heart, with personal relationships that revolves entirely around their needs, time and convenience. With such attitudes, doctors will likely be able to actively engage consumers in managing their own health.

## HealthCity and SmartHealth Technology in Forefront of the New Paradigm of Healthcare

In order to address the health-centric paradigm, a new initiative called HealthCity has recently been implemented in Kazakhstan. 21 It aims to create an integrated network of private technology-driven and patient-centered primary care clinics and diagnostic center to serve communities’ healthcare needs at highest international standards. To ensure quality in its infrastructure, the HealthCity project established partnerships with such global technology companies as Philips and Medtronic. HealthCity facilities will have online medical records and telemedicine activities, home health, community systems, among other which allow supporting a growing community of 50,000 to 100,000 people in key cities of Kazakhstan where HealthCity will operate. Overall, activities of HealthCity lead to transformation of Kazakhstan’s healthcare system.

In order to empower primary care physicians and customers of its clinics, the HealthCity project will employ a proprietary application called SmartHealth comprising of three main components:

1. Diagnostic algorithms «Symptoms Online», designed for web and mobile platforms in three languages: Kazakh, Russian, and English;
2. Web resources of zdrav.kz web-portal with many years of successful track record of providing Internet users with widely available information as how to maintain health and treat illnesses; and,
3. Electronic search system helping to locate specialized doctors and healthcare organizations.

At the heart of SmartHealth there are more than 70 algorithms integrated together as the “Symptoms Online” product. They represent a logical process as how to interpret symptoms of many diseases allowing users to make informed decisions. These algorithms are presented in the form of web and mobile applications for Android and iOS integrated with zdrav.kz portal containing a wealth of information about thousands of diseases and conditions, as well as about methods for their prevention, early detection and treatment.

Because of availability of such resources, SmartHealth users will be able to solve many common medical problems that do not require the participation of the medical professional or visit a medical institution. In more complex cases SmartHealth provides opportunities for informed and targeted search for assistance of a general practitioner or a specialist. In such efforts the users may be able to utilize SmartHealth resources allowing them to find needed healthcare organizations and medical professionals.

This HealthCity project will significantly reduce the burden on the health care system, promoting active participation of citizens and empowering them to make decisions about their own health. SmartHealth - is a technology solution that aims at providing accessibility and convenience in obtaining knowledge about medicine.

## Summary

In summary, the current healthcare paradigm in Kazakhstan and many countries around the world is centered around hospitals and highly specialized medical doctors. It can be described as disease-centric medicine, dominated by technology and aggressive treatments. An alternative to this is an alliance of primary care doctors with consumers, empowered and engaged to be active participants in healthcare decisions on both sides. Only by building such an effective and fully potential ecosystemic healthcare system will we be able to successfully prevent diseases and achieve higher quality and quantity of life for our citizens. By building a healthier and brighter healthcare ecosystem, we have a great opportunity to improve quality of life locally and globally.
